# A Compact Wideband Vivaldi Antenna for Non-Invasive Glucose Monitoring

**DOI:** 10.3390/mi15111389

**Published:** 2024-11-16

**Authors:** Shasha Yang, Yu Wang, Shiwen Gao, Yi Zhuang, Lifeng Wang, Zhenxiang Yi, Weixun Zhang

**Affiliations:** 1 Key Laboratory of MEMS of the Ministry of Education, Southeast University, Nanjing 210096, China; 230228390@seu.edu.cn (S.Y.); 213202894@seu.edu.cn (Y.W.); 213220700@seu.edu.cn (S.G.); 213223805@seu.edu.cn (Y.Z.); xp@seu.edu.cn (Z.Y.); 2 China Southern Power Grid Company Limited, Guangzhou 510000, China; zhangwx5@csg.cn

**Keywords:** Vivaldi antenna, glucose level, non-destructive detection, linear regression

## Abstract

Due to the high gain, wide bandwidth, and directional radiation characteristics of Vivaldi antennas, this paper conducted relevant research on the feasibility of non-destructive blood glucose detection based on Vivaldi antennas. The research included finite element method (FEM) simulation and glucose concentration monitoring. In the simulation stage, the power transmission and reflection characteristics, radiation characteristics, and electric field distribution characteristics of the antenna were described in detail. In the test stage, the S_11_ response of the antenna to variation in glucose concentration in the range of 0–6.11 mg/mL was measured, including the S_11_ amplitude and phase. The experimental results show that there is a high linear correlation between the S_11_ response and glucose concentration, and the sensitivity of the S_11_ amplitude response to the variation in glucose concentration is close to 0.3445 (dB/(mg/mL)) at 14.2556 GHz, and the sensitivity of the S_11_ phase response to the variation in glucose concentration is about 0.5652 (degree/(mg/mL)) at 14.37 GHz. In addition, the predicted results of the glucose concentration based on linear regression are discussed.

## 1. Introduction

With the improvement in productivity and the development of society, abundant resources have greatly met people’s living needs. However, unhealthy and unreasonable dietary structures and lifestyles have also brought a series of disease troubles to contemporary people. As a common disease, the number of diabetes patients is increasing year by year. According to the International Diabetes Federation, 463 million people worldwide had diabetes by 2019, and it is expected to grow to 700 million by 2045 [1]. Since preventive treatment and cure methods for diabetes have not yet been developed, regular blood glucose monitoring is the most effective way to control the blood glucose level (BGL) [2].

The commonly used blood glucose monitoring (BGM) methods in clinical practice mainly obtain blood glucose values by collecting venous or fingertip nerve endings for in vitro analysis. Although this invasive BGM method has high accuracy, long-term continuous blood sampling will increase the risk of blood cross-infection, and will cause physical pain and psychological pressure to patients [3]. At the same time, this method is not suitable for continuous glucose monitoring (CGM). In addition, a minimally invasive micro-needle array detection method which can be used for continuous blood glucose monitoring has gradually become popular in recent years, and interstitial fluid (ISF) is extracted by micro-needles to achieve the purpose of BGM [4]. However, the lag of the glucose level (GL) in ISF will affect the accuracy of CGM [5], while prolonged wearing will still cause discomfort and allergic reactions in patients. Therefore, there is great significance in studying a low-cost, high-precision non-destructive BGM method for alleviating the pain of patients and improving their treatment methods.

At present, the commonly utilized non-destructive BGM methods are divided into optical methods and non-optical methods. According to the different wavelengths and action mechanisms, optical methods can be divided into near/middle infrared spectroscopy [6,7], optical rotation [8], Raman spectroscopy [9], and other optical methods. An optical non-destructive BGM method has the advantages of high resolution and strong penetration, but the disadvantages are a weak signal and easy interference. Non-optical methods include blood substitute detection [10,11], reverse ion electroosmosis [12,13], and microwave detection [14,15]. The blood substitute detection method lacks theoretical support and accuracy due to the unclear correlation between glucose content in substitutes and blood glucose. The ISF extravasation method can cause damage to the skin and has a serious time lag. The non-destructive detection of the blood glucose level using microwave technology has been a research focus in recent years. The electromagnetic wave with a working frequency of 300 MHz−300 GHz is deeply transmitted into human tissues. The reflected wave and the transmitted wave will change in phase and amplitude due to the change in blood glucose concentration, and the blood sugar level can be determined by measuring the change in S parameters [16]. A recent study analyzed and summarized the electromagnetic sensors used for non-destructive BGM in recent years [17]. In the research of microwave devices, changes in blood glucose levels are reflected by measuring S parameters, including the resonant frequency, S_11_, and S_21_. In references [18,19], microwave resonators were used for the non-destructive detection of blood glucose. Due to the influence of dispersion, the change in permittivity caused by variation in the blood glucose concentration can lead to a shift in the resonant frequency, and it has been experimentally proven that the resonant frequency is linearly related to glucose concentration. In references [20,21], the amplitude of the S parameter was selected as the measurement value for detecting variations in glucose concentration. According to the existing research, the BGM measurement based on microwave technology can be applied to develop a miniaturized system, which can realize CGM under the condition of ensuring human safety and has great development prospects for the research field of wearable medical devices.

The Vivaldi antenna is a wideband, high-gain antenna structure that can be used in fields such as microwave imaging [22], radar [23], and communication [24]. In the biomedical field, Vivaldi antennas are widely used in the design of biosensors. Meanwhile, the Vivaldi antenna also has the advantages of a simple structure, easy preparation, and easy integration. This paper introduces a non-destructive GL research method based on a Vivaldi antenna for the first time. The Vivaldi antenna can capture the weak signal changes caused by changes in the GL due to the characteristics of a wide bandwidth, strong anti-interference ability, high gain, and low ripple [25]. The antenna was modeled and simulated in HFSS software, and relevant information such as the S parameters and electric field distribution were obtained. By using the terminal emission characteristics of the Vivaldi antenna, the glucose solution was placed in a suitable position for the concentration experiment. The experimental results of the S parameters preliminarily indicate the feasibility of the non-destructive detection of the GL based on a Vivaldi antenna.

## 2. Design and Simulation

### 2.1. Design of Vivaldi Antenna

**Figure 1:**
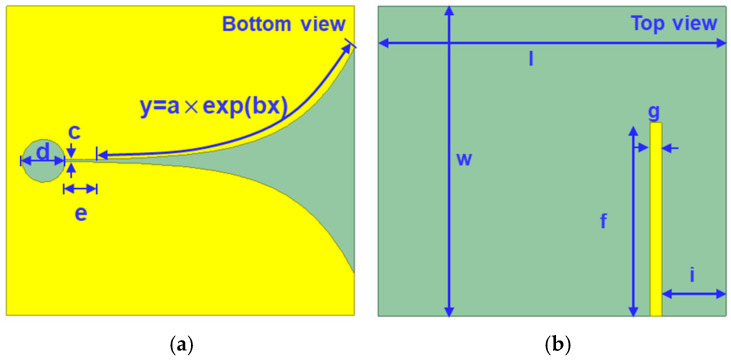
Structure of the Vivaldi antenna: (**a**) bottom view and (**b**) top view.

According to different application requirements, the Vivaldi antenna has derived many different variants. This paper focuses on the research of a compact wideband coplanar Vivaldi antenna (C-CVA). The Vivaldi antenna mainly includes three parts: a dielectric substrate, feed structure, and radiation structure. In Figure 1, the radiation structure is composed of a circular resonant cavity, a rectangular slot line, and an exponential gradient line, while the feed structure is composed of a microstrip line. The microstrip line is coupled to the slot line through the dielectric substrate. The antenna was designed using Rogers Ro4350B with a dielectric constant of 3.48, dielectric loss tangent of 0.0037, and thickness of 0.76 mm as the substrate, and the copper-clad thickness was 0.035 mm. The structural parameters of the Vivaldi antenna are listed in Table 1.

### 2.2. FEM Simulation

**Figure 2:**
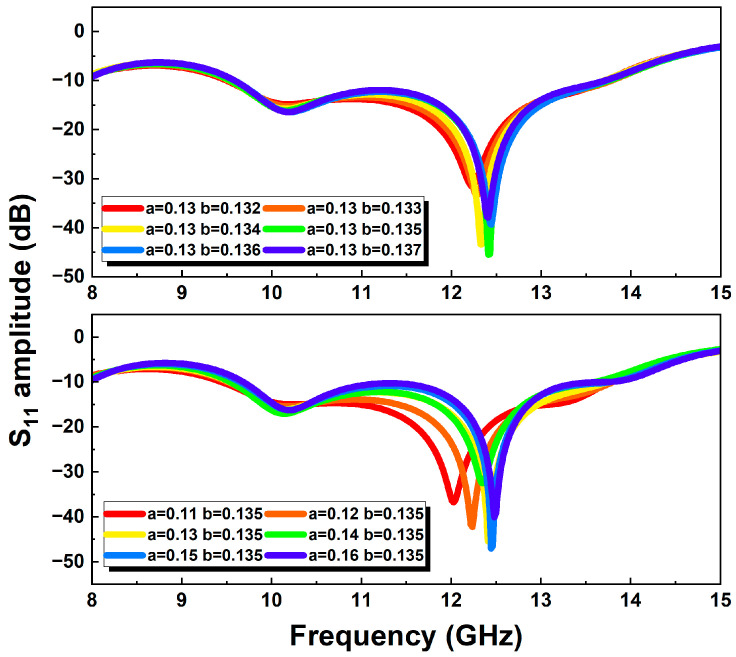
Parameter optimization results in HFSS.

**Figure 3:**
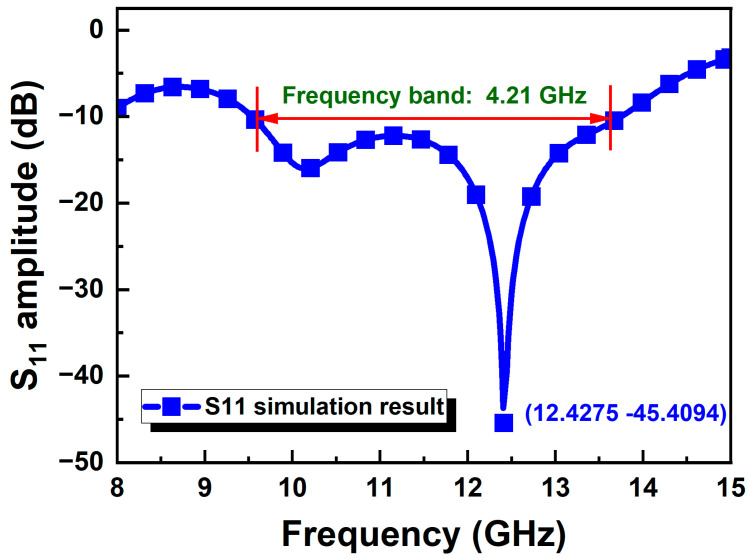
Simulation result of the reflection coefficient for the proposed Vivaldi antenna.

**Figure 4:**
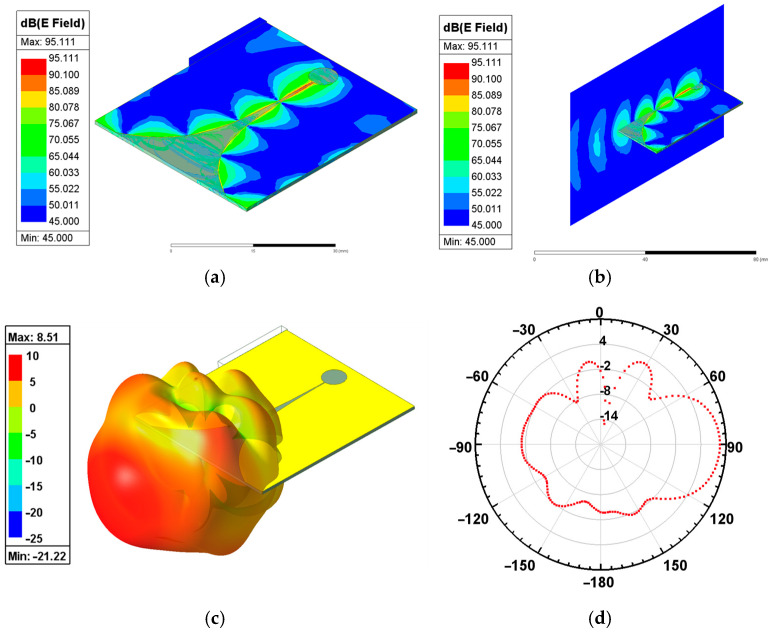
FEM simulation of the Vivaldi antenna. (**a**) Surface electric field distribution; (**b**) spatial electric field distribution; (**c**) three-dimensional radiation pattern, and (**d**) vertical lobe diagram.

The Vivaldi antenna was simulated in the frequency range of 8–15 GHz with a center frequency point of 12 GHz. The structural design of the antenna was mainly based on the parameterized scanning function of the HFSS simulation software. The geometric size parameters of the Vivaldi antenna are shown in Figure 1, and they were optimized to obtain the final size of the antenna. Among them, a and b are the dominant parameters of the exponential gradient slot line, which play a guiding role in the transmission of antenna signals and energy radiation. The partial parameterized scanning results of parameters a and b are shown in Figure 2. The simulation results of the S_11_ amplitude in the figure show that the changes in parameters a and b will affect the work frequency band and return loss of the antenna. When a does not change with b, the opening depth of the exponential gradient line is basically fixed, while the area near the end of the opening of the exponential gradient line changes greatly. Therefore, the low-frequency characteristics of the antenna are greatly affected. When b does not change with a, the opening of the exponential gradient line shows an expanding trend with the increase in a, and the low-frequency characteristics, high-frequency characteristics, and work frequency band of the antenna change. The simulation results of the antenna reflection coefficient S_11_ after parameter optimization are shown in Figure 3. Taking the return loss of −10 dB as the standard to measure the transmission efficiency of the antenna, the working frequency band of the Vivaldi antenna is over the frequency band of 9.54–13.75 GHz (S_11_ < −10 dB), and it shows good resonance at 12.43 GHz. Figure 4a shows the surface electric field distribution of the antenna at the frequency point of 12 GHz. The antenna is fed by exciting a microstrip transmission line, which is coupled to the rectangular slot line of the Vivaldi antenna radiation structure through the dielectric substrate. The electric field flows from the beginning to the end of the exponentially open slot line, and a strong electric field distribution is generated at the edge of the slot line. Figure 4b shows the spatial electric field distribution of the antenna, which illustrates the propagation of the electric field along the central axis of the antenna towards space. Figure 4c shows the three-dimensional radiation pattern of the antenna at 12 GHz, describing the radiation power density of the Vivaldi antenna in different directions. The main lobe of the antenna points to the end of the opening of the exponentially graded slot line, that is, the maximum radiation direction of the antenna, and the maximum gain of the antenna reaches 8.51 dBi. Figure 4d shows the vertical lobe diagram of the antenna (phi = 0 degree), from which it can be seen that the Vivaldi antenna has extremely strong directionality.

### 2.3. Principle of GL Detection

The non-destructive detection of the BGL based on an electromagnetic sensor is realized by the near-field radiation of the antenna. The measured object is placed in the direction of the antenna’s electromagnetic radiation, and the electromagnetic wave penetrates through the outer layer and reaches the inside of the blood. The S_11_ parameter of the antenna can be calculated as

(1) $$S_{11}=\frac{z_{L}\left(\varepsilon_{GL}\right)-z_{0}}{z_{L}\left(\varepsilon_{GL}\right)+z_{0}}$$

where $z_{0}$ is the incident impedance and $z_{L}\left(\varepsilon_{GL}\right)$ is the reflected impedance of the antenna, respectively. In Equation (1), the reflected impedance $z_{L}\left(\varepsilon_{GL}\right)$ is related to the dielectric coefficient of the blood $\varepsilon_{GL}$, which in turn is related to the GL of the blood. Therefore, the GL of the blood will eventually lead to a change in the amplitude and phase of the S_11_ parameters. In another word, the GL can be predicted by testing the S_11_ parameters of the antenna.

## 3. Measurements and Discussions

### 3.1. Test Platform

**Figure 5:**
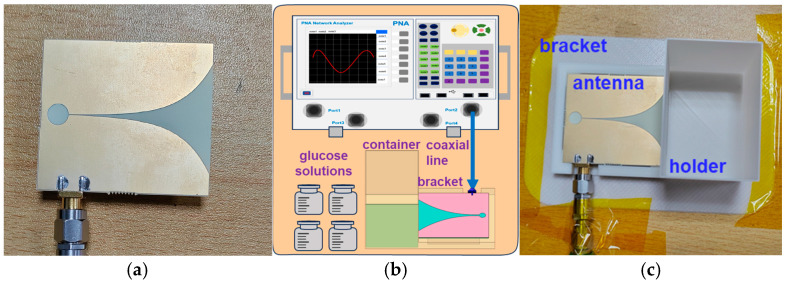
(**a**) Photograph of the fabricated Vivaldi antenna, (**b**) setup of the GL experiment, and (**c**) photograph of the GL experiment.

The S-parameter measurement of the antenna was completed by using the vector network analyzer (VNA) (Figure 5b), which was connected to the antenna through a coaxial transmission line. Based on the end-emitting characteristics of the Vivaldi antenna, the glucose solution was placed at the end of the antenna. Therefore, a bracket was specially designed for testing, as shown in Figure 5c. The front half of the bracket was designed with a groove for fixing the antenna, and the container in the back half was designed to hold the glucose solution. The test bracket was made of resin material using 3D printing technology, and there were no issues such as liquid leakage.

### 3.2. Measurement of GL

**Figure 6:**
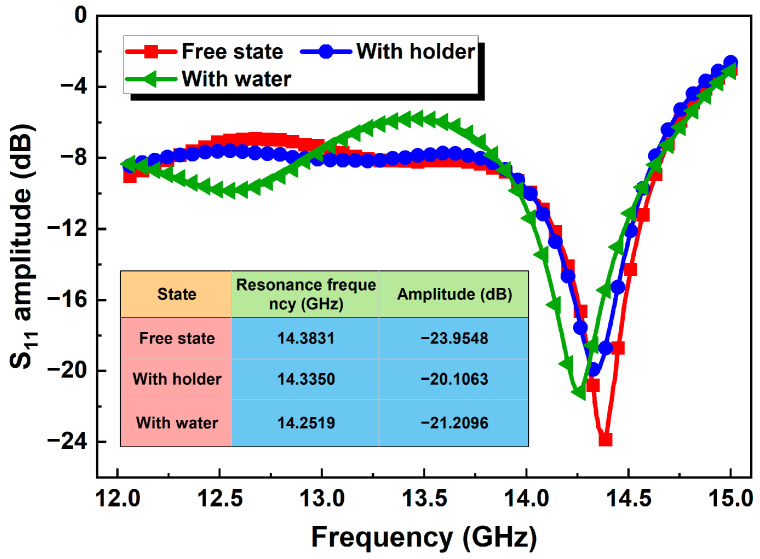
Measured amplitude of S_11_ for the Vivaldi antenna under different conditions.

In order to reduce the error, the experiment was conducted in a safe, stable, and undisturbed environment. In Figure 6, the performance of the antenna under three different conditions was tested and evaluated: free state, fixed on the test bracket, and with deionized water in the container. The reflection coefficient S_11_ of the antenna underwent varying degrees of frequency shift and amplitude change in the three different states. Specifically, the antenna was observed to have a deep resonance at 14.3831 GHz in its free state. When the antenna was fixed on the test bracket, the resonance frequency shifted down by approximately 0.0481 GHz. Similarly, when the test container was filled with deionized water, the resonance frequency of the antenna shifted down to 14.2519 GHz. However, the intervention of the test bracket and the solution does not have a significant impact on the matching of the antenna. The radiation energy of the antenna is not affected by the strong reflection of the incident wave when transmitted to the solution, and can still maintain good performance.

In the experiment of the antenna’s response to glucose variation, deionized water/glucose solution was used to study the sensitivity to the GL’s variation. During the experiment, the antenna was connected according to the method shown in Figure 5 and fixed on the insulated experimental table with tape to prevent mechanical displacement and vibration from affecting the experimental results. The initial GL of deionized water was 0 mg/mL, and the container was filled with 20 mL of deionized water. During each measurement, a dropper was used to remove 2 mL of solution from the deionized water and 2 mL of a pre-prepared glucose solution was added to the deionized water. Then, the deionized water/glucose solution was stirred gently to ensure the uniform mixing of the solution. Hence, the glucose level can be changed while maintaining the same solution volume in this way, and the preparation method of the glucose solution is shown in Table 2. Compared to directly adding glucose to the solution, this method can avoid experimental errors caused by incomplete solution mixing effectively.

**Figure 7:**
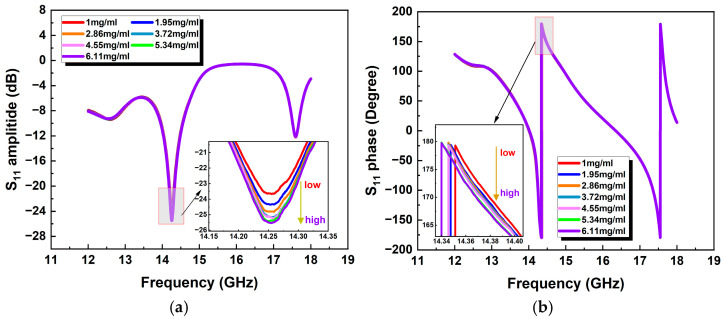
Measured return loss S_11_ for the antenna, varying with the glucose level. (**a**) Amplitude of S_11_ and (**b**) phase of S_11_.

**Figure 8:**
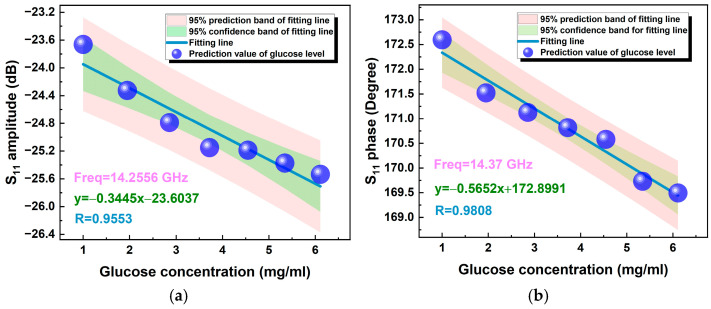
(**a**) The fitted line of the S_11_ amplitude, varying with the GL, and (**b**) the fitting of the S_11_ phase, varying with the GL.

As shown in Table 2, the glucose concentration varied from 0 to 6.11 mg/mL, which can cover the range from hypoglycemia to hyperglycemia. For the glucose concentration at each stage, the complex reflection coefficient S_11_ (phase and amplitude) of the antenna was recorded over the frequency band of 12–18 GHz by using the VNA, and the results are plotted in Figure 7. As shown in the figure, the performance of the antenna remained stable during the experiment, and within a specific frequency band, the antenna’s S_11_ response showed a high sensitivity to variation in GLs. Selecting appropriate frequency points to linearly fit the S_11_ response with the reference GLs, the results are shown in Figure 8. Obviously, the S-parameter response of the sensor has a high correlation with the reference GL. At 14.2556 GHz, the correlation coefficient R of the S_11_ amplitude of the sensor is 0.9553, while the correlation coefficient R of the S_11_ phase of the sensor is 0.9808 at 14.37 GHz. It is worth noting that the S-parameter response of the sensor at multiple frequency points exhibits high sensitivity to GL variation. This paper only studies the tested results at the single frequency point with the highest sensitivity.

**Figure 9:**
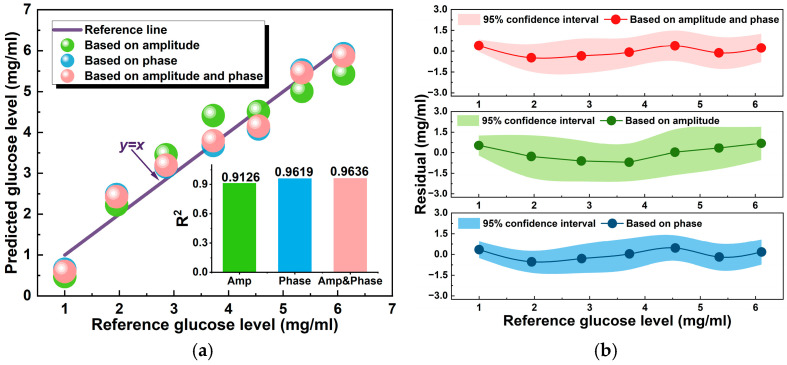
(**a**) Predicted GL results based on the S_11_ response and (**b**) the residual analysis.

Linear regression and Gaussian process regression (GPR) are two commonly used regression analysis methods; both regression models have their own merits and demerits, and are suitable for different scenarios. GPR is usually suitable for low-dimensional and small-sample regression problems. However, its algorithm is complex, and the computational cost is high. Linear regression is one of the most basic regression models, and its advantages include the following: (i) the model is simple to use and easy to implement; (ii) it is suitable for large-scale datasets with high computational efficiency. Considering there is a strong linear correlation (R > 0.95) between the S_11_ parameter response and glucose concentration and the linear regression model has the advantages of a simple algorithm and high efficiency, here we use a linear regression algorithm to analyze and explain the predicted results of the glucose level. Firstly, the glucose level was predicted by linear regression according to the amplitude and phase response of the S_11_. Figure 9a contains the predicted results of the GL based on the S_11_ amplitude response and S_11_ phase response, respectively. In addition, it also contains the GL prediction result obtained by combining the amplitude and phase responses of the antenna with the binary linear regression model. In order to evaluate the accuracy of the prediction model, the reference GL was compared with the predicted GL, and it can be seen that the predicted value of the GL is closely related to the reference value. Compared with the univariate regression prediction model, the multivariate regression prediction model exhibits a higher determination coefficient R^2^, better fitting performance, and higher prediction accuracy and reliability. In this paper, the root mean square error (RMSE) and mean absolute error (MAE) of the prediction are used as performance criteria for evaluating the model. Specifically, for the S_11_ amplitude response, the RMSE and MAE are about 0.503 mg/mL and 0.4496 mg/mL, respectively. For the S_11_ phase response, the RMSE and MAE are close to 0.3322 mg/mL and 0.2915 mg/mL, respectively. For the binary linear regression prediction model based on phase and amplitude responses, the RMSE and MAE are close to 0.3248 mg/mL and 0.2932 mg/mL, respectively. Moreover, Figure 9b shows the residual analysis of the three kinds of glucose level prediction models. The 95% confidence interval provides an estimate of a probability sample and its possible error range, which is used to estimate the accuracy and reliability of the model. It shows that the residual confidence intervals of the three predicted results are distributed near zero and there are no obvious abnormal values. Among them, the prediction model based on the S_11_ amplitude response has relatively low accuracy.

Table 3 lists the comparison between relevant studies and this work on blood glucose non-destructive testing. Firstly, this work is based on the Vivaldi antenna for the non-destructive detection of blood glucose, and its excellent end emission characteristics enable the sensor to achieve high sensitivity. Secondly, it can be seen that most studies tend to focus on single measurement parameters, while this work is about multiple measurement parameters. The advantage of this approach is that multiple measurement parameters make the reference data selective and the prediction accuracy of BGLs higher.

## 4. Conclusions

This paper has carried out preliminary research on GL detection using Vivaldi antennas for the first time. The antenna was optimized by HFSS simulation software, and the electric field distribution and end emission characteristics of the antenna were explained. In the response test of the antenna to the variation in glucose concentration, the S-parameter responses of the antenna varying with different concentrations were obtained, and the experimental results were explained and discussed. Finally, based on the original response results of the antenna, the GL was predicted using a linear regression model, and it was confirmed that the multiparameter predicted results are more reliable than the single parameter. This paper provides a new method and idea for non-destructive blood glucose detection.

## Figures and Tables

**Table 1:** Structural parameters of the Vivaldi antenna in this paper.

Parameter	Description	Value (mm)
a	/	0.13
b	/	0.135
c	Width of rectangular slot	0.26
d	Diameter of circular resonant cavity	5.6
e	Length of rectangular slot	2.4
f	Length of microstrip line	25
g	Width of microstrip line	1.5
i	/	8.3
w	Width of substrate	40
l	Length of substrate	45

**Table 2:** Solution preparation instructions based on equal volume displacement method.

Prepared Solution Concentration (mg/mL)	Current Solution Concentration (mg/mL)	Test Solution Concentration (mg/mL)
0.0	0.00	0.00
10.0	0.00	1.00
10.5	1.00	1.95
11.0	1.95	2.86
11.5	2.86	3.72
12.0	3.72	4.55
12.5	4.55	5.34
13.0	5.34	6.11

**Table 3:** Performance comparison of sensors based on blood glucose detection.

Ref.	Sensor Type	Frequency (GHz)	Measured Parameter	Sensitivity
[18]	Rectangular meandered line resonator	9.2	Resonant frequency of S_21_	1.08 MHz/(mg/dL)
[19]	Microstrip antenna	1.9	Resonant frequency of S_11_	0.3 MHz/(mg/dL)
[20]	Ultrawideband microwave antenna	6.5	S_21_ amplitude	S_21_ amplitude changes by 6 dB within the range of blood glucose concentration 0–40 mg/mL
[21]	Dual microwave complementary split ring resonators	2.42	S_21_ amplitude	0.008 dB/(mg/mL)
This work	Vivaldi antenna	14.2556/14.37	S_11_ amplitude and phase	0.3445 dB/(mg/mL) 0.5652 Deg/(mg/mL)

## Data Availability

The original contributions presented in the study are included in the article, further inquiries can be directed to the corresponding author.
